# Impact of Exopolysaccharides from *Fructilactobacillus sanfranciscensis* Ls5 on the Quality of Mantou: A Comparative Study of Fermentation Conditions

**DOI:** 10.3390/foods13223611

**Published:** 2024-11-12

**Authors:** Juanxia Wang, Huiying Tian, Jiale Wang, Jingjing Liang, Jiao Li, Luciana Jimenez, Pascal Lejeune, Shiwei Zhou, Guohua Zhang

**Affiliations:** 1 School of Life Science, Shanxi University, Taiyuan 030006, China; 18636548656@163.com (J.W.); 15536386811@163.com (H.T.); 17835170325@163.com (J.W.); jingjingliang0322@163.com (J.L.); lijiao@sxu.edu.cn (J.L.); 2 Lesaffre Institute of Science and Technology, 101 Rue de Menin, 59700 Marc-en-Baroeul, France; l.jimenez@lesaffre.com (L.J.); p.lejeune@lesaffre.com (P.L.); 3 Lesaffre Management (Shanghai) Co., Ltd., Shanghai 200235, China

**Keywords:** sourdough, EPS, Mantou, shelf-life, in vitro digestion

## Abstract

Lactic acid bacteria (LAB) and their exopolysaccharides (EPS) have the potential to enhance the quality of flour-based products. This study investigated the effect of EPS produced by *Fructilactobacillus sanfranciscensis* Ls5 on the quality of Mantou. LAB strains with high EPS production were isolated from traditional fermenters, and their characteristics and EPS properties were examined. Four different fermentation conditions (Blank, Yeast, Ls5-Yeast, and EPS-Yeast) were compared in terms of their effects on the physicochemical properties, in vitro digestion characteristics, and shelf-life of Mantou. The incorporation of Ls5 and its EPS into Mantou resulted in a lower dough fermentation pH, increased organic acid production, and enhanced fermentation activity. Additionally, the incorporation of Ls5 and its EPS led to significant improvements in the quality of the Mantou, including the extension of shelf-life, improved sensory evaluation, and a reduction in the sugar content. Additionally, there was an increase in resistant starch content during digestion in both types of Mantou, which could offer potential benefits to human glycemic health.

## 1. Introduction

Sourdough is a mixture of processed grains fermented by LAB and yeast products combined with water [1]. Although active dry yeast is currently the primary microorganism used for fermenting flour-based products, it has several drawbacks compared to conventional mixed bacterial starters. Active dry yeast offers a singular taste, lacks sufficient flavor, exhibits poor overall sensory quality, and has limited re-steaming capability. Mantou is a traditional Chinese fermented food. It is mainly made from wheat flour, water, and either yeast or sourdough. The ingredients are fully mixed, fermented, and steamed. Northern China is the main consumer market for Mantou, accounting for about 40% of the total wheat product consumption [2]. During the production of sourdough Mantou, LAB and yeast are fully fermented, giving Mantou a unique flavor and delicate taste. Compared to baked bread, Mantou is produced at a lower manufacturing temperature, resulting in less content of Maillard reaction products. Therefore, the popularity of Mantou in the global market continues to rise due to its health benefits.

LAB isolated from sourdough can generate EPS during fermentation [3]. EPS has various benefits on fermented flour products, such as increasing the volume of pasta products, retarding their aging process, enhancing water absorption, improving the rheological properties of dough, slowing down starch regrowth, stabilizing freeze-thaw, boosting dough viscosity, and reducing dough hardness [4,5,6,7]. Zhang et al. [3] found that *Lactobacillus sanfranciscensis* 1001 exhibits high EPS production, has the ability to decrease the moisture content of Mantou, enhance its specific volume, and significantly enhance the sensory attributes of the Mantou. Cagno et al. [8] added EPS-producing *L. plantarum* and *Weissella cibaria* to the dough and observed that the viscosity of sourdough increased, its hardness decreased, and the specific volume of the product increased significantly compared to the normal dough. Furthermore, Sandra et al. [9] found that adding glucan alone to the bread in the control group while adding EPS-producing Lactobacillus spp. to bread in the experimental group resulted in Lactobacillus + EPS bread having a greater specific volume and reduced hardness and aging rate.

In this study, we focused on the EPS produced by LAB and their roles in the fermentation process of sourdough and revealed their potential impact on the quality and characteristics of Mantou. We compared the impact of different fermentation conditions (Blank, Yeast, Ls5-Yeast, and EPS-Yeast groups) on the physicochemical properties, in vitro digestion characteristics, and shelf-life of Mantou. The Ls5-Yeast group emphasized the synergistic effect of LAB and yeast, while the EPS-Yeast group focused on how the exopolysaccharide produced by LAB affected the physical properties and fermentation effects of sourdough. The combination of exopolysaccharides of LAB and yeast was systematically studied to explore its effect on the quality of Mantou, providing new ideas for the fermentation process. The ultimate goal of this study was to improve the industrialization of traditional fermented flour products and provide consumers with high-quality, nutrient-dense, traditional fermented pasta options.

## 2. Materials and Methods

### 2.1. Materials

A total of 52 LAB (LAB) strains were obtained from Chinese Mantou and provided by the College of Life Science, Shanxi University, Taiyuan, China. These strains were stored at −80 °C for further use. A total of 50 μL [10] of activated LAB were inoculated into 5 mL of modified mMRS liquid medium [11] and cultured at 30 °C for 24 h in the incubator to obtain the LAB fermentation solution.

### 2.2. Extraction and Determination of EPS

Taking the above fermentation solution, the crude EPS solution was extracted according to the method by Zhang G et al. According to the phenol-sulfuric acid method, 2 mL of crude EPS solution was measured for OD490 to calculate the EPS production.

### 2.3. Comparison of Characteristics of Two Strains

The cell dry weight method was employed for the determination [12]. The LAB fermentation solution was centrifuged at 9000× *g* for 15 min. The cells obtained were washed twice with deionized water, transferred to a pre-weighed centrifuge, and dried at 90 °C to a constant weight, and the value was recorded as the amount of growth. A total of 100 μL of activated LAB were inoculated into 5 mL of mMRS medium, cultured at 30 °C, and sampled at 2 h intervals to determine the OD600, generating the growth curves of the strain. The bacterial fluid was spread thinly, followed by a dropwise addition of crystal violet staining solution. After 2 min, the residual water was rinsed off with an iodine solution, followed by covering the bacteria with an iodine solution for 1 min. Next, 95% alcohol was applied for 30 s, rinsed with red solution for 2 min, and then washed. The stains were then rinsed with 95% alcohol for 30 s. The cells were then re-stained with red for 2 min and washed again.

### 2.4. Comparison of EPS Characteristics of Two Strains

#### 2.4.1. Isolation and Purification of EPS

The crude EPS obtained in Section 2.2 was prepared as a 10 mg/mL solution in Tris-HCl buffer, filtered, and then concentrated. The sample was then subjected to chromatography using a gradient of Tris-HCl buffer containing varying concentrations of NaCl. The main components of the EPS were isolated, with Ls5-EPS eluents collected in fractions #12–20, 79–84, and 85–89, and Fs7-EPS eluents collected in fractions #70–90. The flow rate of the peristaltic pump was set to 3 mL/min, and a centrifugal tube was collected for every 5 mL of the eluent. The sugar content was quantified using the method described in Section 2.2. The components were concentrated, dialyzed, and lyophilized for use in the subsequent experiments.

#### 2.4.2. Determination of EPS by Ultraviolet Spectrum

EPS was prepared in a solution of a specific concentration with distilled water and then scanned in the ultraviolet spectrum within the range of 190–350 nm to assess purity. If the sample curve exhibits absorption peaks at 260 nm and 280 nm, it indicates the presence of nucleic acids and proteins, signifying low sample purity. Conversely, if the sample curve is smooth and flat without distinct peaks, it indicates low levels of nucleic acids and proteins, signifying high sample purity.

#### 2.4.3. Determination of Monosaccharide Composition and Molecular Mass

High-performance anion-exchange chromatography was performed and HPSEC–MALLS analyses were conducted according to the method described by Yufei Ji [13].

### 2.5. Determination of pH

Determination of pH of LAB Fermentation Solution: Activated LAB (50 μL) were inoculated into 5 mL mMRS medium and incubated at 30 °C for 2 days. Samples were collected at 2 h intervals. The pH values at each sampling point were measured.

Determination of pH of sourdough and Mantou: The samples of sourdough or Mantou were roughly chopped into pieces smaller than 1 cm^3^. After chopping, 10 g of the crushed sample was placed into a mortar, along with 100 mL of distilled water, which had been boiled and cooled prior to use. This mixture was then crushed into a uniform paste with the pestle before being transferred to a beaker for measurement of pH according to the method by Dong M et al. [14] every two hours.

### 2.6. Determination of TTA (Total Titrable Acids)

Determination of TTA of LAB Fermentation Solution: A total of 10 mL of fermentation solution was mixed with 50 mL of distilled water. The mixture was then titrated with 0.1 mol/L NaOH solution, and the volume was consumed until a pH of 8.2 was recorded [15]. The calculation formula is as follows:

(1) $$\mathrm{TTA}\left(g/L\right)=\frac{{\mathrm{CVKV}}_{0}}{{\mathrm{mV}}_{1}}$$

where C is the concentration of the standard NaOH solution (mol/L), K is the conversion coefficient (0.090 for lactic acid), V_0_ is the total volume of the sample solution (mL), V is the volume of NaOH consumed (mL), m is the volume of the sample (mL), and V_1_ is the volume of the sample obtained by titration.

Determination of TTA of sourdough: The sourdough liquid was titrated with a 0.1 mol/L NaOH solution to reach a pH of 8.2. The TTA value of sourdough was determined using the specified formula:

(2) $$X=\frac{0.1\times (V_{1}-V_{2})\times 0.090}{10}\times 1000$$

where V_1_ represents the volume of the NaOH solution consumed by the experimental group, and V_2_ is the volume of the NaOH solution consumed by the Blank group.

### 2.7. Preparation of Mantou

In the Blank group, 25 mL of water was combined with 50 g of flour and stirred in a mixer for 10 min. The dough was then pressed 5–7 times using a dough press and manually formed into balls, which were placed in a rising box at 32 °C with 70% humidity for 1 h. Subsequently, the dough was steamed in cold water for 20 min.

The Yeast group followed the same procedure as the Blank group, except that yeast powder (0.5 g) was added before mixing with 50 g of flour.

The Ls5-Yeast group was supplemented with 50 mL of cultured Ls5 fermentation liquid to prepare the Yeast group, with its density adjusted to 10^9^ CFU/mL. The bacterial slurry was centrifuged, and an equal amount of raw material was added to the Yeast group. Preparation was performed under the same conditions as those used for the Blank group.

The EPS-Yeast group was mixed with an equal amount of material from the Yeast group and 0.2% EPS per volume of flour, following the conditions of the Blank group.

### 2.8. Determination of Specific Volume, Moisture Content, Fermentation Capacity

The specific volume was measured according to the method by Dong M et al. [14].

The moisture content was measured by the direct drying method according to the method by Dong M et al. [14].

The fermentation capacity was measured according to the method by Teng C et al. [16].

### 2.9. Determination of Microorganisms

After cooling the four kinds of Mantou for 1 h, three Mantou samples were put into a sealed bag and stored at room temperature (20 ± 2 °C), and the indices were measured every 12 h. At the same time, Mantou was stored in the refrigerator at 4 °C, and the indexes were measured every 2 days. The total colony count, mold, and yeast were detected according to GB 4789.2-2022 [17] and GB 4789.15-2016 [18], respectively.

### 2.10. In Vitro Starch Digestion Study

#### 2.10.1. In Vitro Starch Digestion

The in vitro digestive solution was formulated according to the method described by Brodkorb [19], which includes simulated saliva (SSF), simulated gastric fluid (SGF), and simulated intestinal fluid (SIF) in mmol/L. Various Mantou samples were ground for 3 min using a grinder, and the resulting crushed Mantou was combined with an equal volume of SSF (containing 75 U/mL alpha-amylase), with the pH adjusted to 7.0 using 6 mol/L hydrochloric acid. The mixture was incubated in a water bath at 37 °C for 2 min to simulate oral digestion. Subsequently, an equal volume of SGF (containing 2000 U/mL pepsin) was added, and the pH was adjusted to 1.5 using 6 mol/L hydrochloric acid. The mixture was then incubated in a water bath at 37 °C for 2 h to simulate gastric digestion. An equal amount of SIF (containing 8 mg/mL bile salts and 3 mg/mL trypsin) was then added and mixed, and the pH was adjusted to 7.2. The mixture was incubated in a water bath at 37 °C for 2 h to simulate the enterodigestive phase. Digestion was terminated by adding 1 mL of food every 20 min and incubating the mixture in a water bath at 95 °C for 5 min. The precipitate was then removed by centrifugation and the OD600 was measured using the DNS method to calculate the reducing sugar content using a standard curve derived from the starch hydrolysis.

#### 2.10.2. Determination of Free Glucose Content (FG) and Total Starch Content (TS)

The samples (1.0 g) were thoroughly mixed with phosphate buffer solution (0.2 mol/L, pH 5.2) and then heated at 95 °C for 20 min. After the temperature dropped to 37 °C, the gelatinized starch samples were centrifuged at 5000 r/min for 10 min. FG was determined according to the method by Singh Sandhu et al. [20] and calculated by the following formula:

(3) $$\mathrm{FG}=\frac{\mathrm{Glucose}\;\mathrm{content}\;\mathrm{in}\;\mathrm{supernatant}}{\mathrm{Quality}\;\mathrm{of}\;\mathrm{Mantou}}\times 100\%$$

A total of 1.0 g of the crushed samples was accurately weighed and placed into a 100 mL triangular flask. Then, 15 mL of distilled water and 10 mL of 6 mol/L HCl were added. The mixtures were then heated and hydrolyzed in a boiling water bath for 30 min before being removed. After cooling, the hydrolysis solution in the triangular flask was neutralized with 6 mol/L NaOH until it turned slightly red. It was then diluted with distilled water to a total volume of 100 mL, filtered, and 10 mL of the filtrate was taken. This filtrate was transferred to a volumetric flask, made up to 100 mL, and thoroughly mixed. TS was determined according to the method by Singh Sandhu et al. [20] and calculated by the following formula:

(4) $$\mathrm{TS}=\frac{(\mathrm{Glucose}\mathrm{content}\;\mathrm{in}\;\mathrm{supernatant}-\mathrm{FG})\times 0.9}{\mathrm{Quality}\;\mathrm{of}\;\mathrm{Mantou}}\times 100\%$$

#### 2.10.3. Determination of Starch Polymerization Degree

The degree of starch polymerization was assessed using the terminal reduction method [21]. A total of 0.50 g of the samples was accurately weighed and thoroughly dissolved in a 2 mol KOH solution. The pH of the solution was then adjusted to neutral using an HCl solution, and the volume was brought up to 50 mL in a volumetric flask. Subsequently, the sample solution was taken, and the reducing sugar content was measured using the DNS method. The degree of starch polymerization in the samples was calculated using the following formula:

(5) $$\mathrm{DP}=\frac{1.1\times W}{50\times G}$$

where W is the Mantou mass (mg), 1.1 is the conversion factor, and G is the reducing sugar content (mg).

#### 2.10.4. Determination of Digestive Starch Type

Following the determination of FG and TS, the quantities of RDS (Rapid-digested Starch), SDS (Slow-digested Starch), and RS (Resistant Starch) were computed using the formula specified in reference [22].

(6) $$\mathrm{RDS}=\frac{(G_{20\min}-\mathrm{FG})\times 0.9}{\mathrm{Quality}\;\mathrm{of}\;\mathrm{Mantou}}\times 100\%$$

(7) $$\mathrm{SDS}=\frac{(G_{120\min}-G_{20\min})\times 0.9}{\mathrm{Quality}\;\mathrm{of}\;\mathrm{Mantou}}\times 100\%$$

(8) $$\mathrm{RS}=\frac{\mathrm{TS}-\mathrm{RDS}-\mathrm{SDS}}{\mathrm{Quality}\;\mathrm{of}\;\mathrm{Mantou}}\times 100\%$$

where G_20min_ is the mass of reducing sugar produced by digestion for 20 min (mg), and G_120min_ is the mass of reducing sugar produced by digestion for 120 min (mg).

### 2.11. Sensory Evaluation

The sensory evaluation methods outlined in the national standard GB/T 35991-2018 [23] and the study by Zhang et al. [24] were adapted to assess the sensory attributes of the Mantou. A group of 10 food engineering majors (comprising 50% male and 50% female participants aged 19–26 years, who fasted for 2 h prior to the experiment) was assembled to conduct a sensory assessment of the Mantou.

### 2.12. Statistical Analysis

Statistical analysis of the data was performed using EXCEL, and SPSS19.0 was used for the significance analysis. Graphs were generated using OriginPro (version 2018).

## 3. Results and Discussion

### 3.1. Screening of LAB with High EPS Production

**Figure 1:**
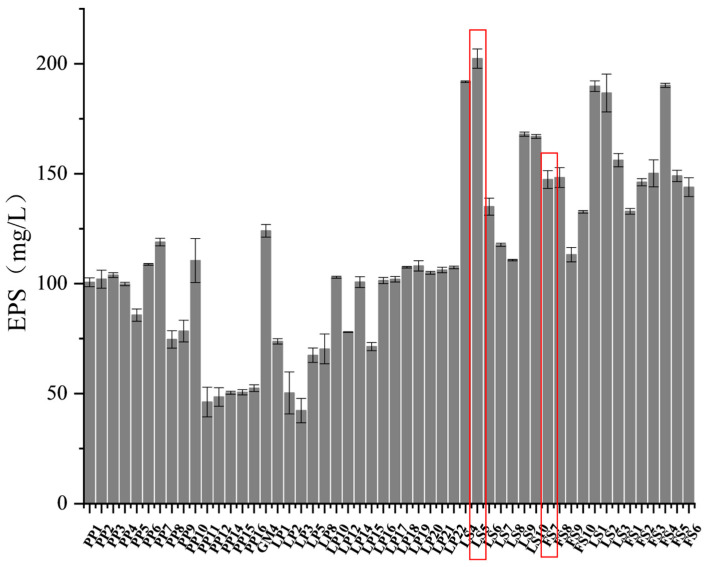
Yield of 52 strains of lactic acid bacteria EPS. The red boxes represent the two selected strains, Ls5 and Fs7.

The screening of 52 Lactobacillus strains for high EPS production is illustrated in Figure 1, which varied from 35.47 to 202.34 mg/L. Among these strains, Ls5 exhibited the highest EPS production, amounting to 202.34 ± 4.40 mg/L. Considering the prevalence of *Fructilactobacillus sanfranciscensis* and *Lactiplantibacillus plantarum* in sourdough, *L. plantarum* Fs7, which demonstrated significant EPS production with an EPS yield of 147.34 ± 4.02 mg/L, was selected for further investigation into strain characterization and EPS properties.

### 3.2. Comparison of Strain Characteristics of the Two Strains

#### 3.2.1. Determination of LAB Growth

**Figure 2:**
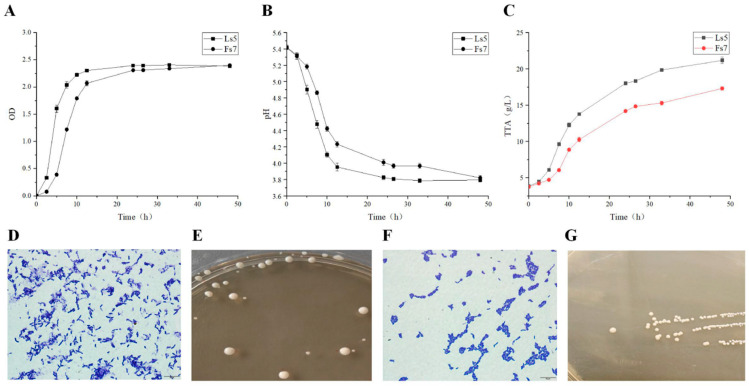
Growth curves of the target strain (**A**), pH of the target strain (**B**), TTA of the target strain (**C**), Morphological characterization of Ls5 (**D**,**E**), Morphological characterization of Fs7 (**F**,**G**).

After 24 h of incubation, the growth of Ls5 was 2.62 ± 0.2 g/L, and the corresponding growth of Fs7 was 2.26 ± 0.2 g/L, as shown in Table 1. A comparison of the data in the table indicates that Ls5 demonstrated superior growth compared to Fs7 under the same incubation period. Figure 2A shows that Ls5 underwent a lag phase (0–2 h), logarithmic phase (2–12 h), and plateau phase (12–48 h) over 48 h. In contrast, Fs7 displayed a lag phase (0–2 h), a logarithmic phase (2–20 h), and a stationary phase (20–48 h) within the same timeframe. Although Ls5 initially exhibited better growth than Fs7, there was minimal disparity in the growth of the two strains after 24 h. Both strains of LAB demonstrated robust growth and rapid reproduction rates. Ls5 achieved an optical density (OD) of 2.41 ± 0.01 at 26.5 h, while *L. plantarum* Fs7 reached an OD of 2.39 ± 0.01 at 48 h. Li et al. [25] reported that the OD of four strains of LAB ranged from 2.0 to 3.0 upon entering the stable phase. Zhang et al. [26] found that the growth yield of *Fructilactobacillus sanfranciscensis* LS-1001 was 2.4 ± 0.1 g/L, with an OD of approximately 2.25 after incubation for 14 h. Li et al. [27] noted that the OD of *L. plantarum* ZJ316 was around 1.8 upon reaching the stable growth stage.

#### 3.2.2. Determination of pH and TTA of LAB Fermentation Solution

In Figure 2B,C, both Ls5 and Fs7 experienced a progressive decrease in pH and a gradual increase in TTA throughout the growth phase. Among the two strains, Ls5 demonstrated a more pronounced reduction in pH and a faster acid production rate. The pH of both strains eventually reached approximately 3.9, with a final acid production of 20.9 ± 0.07 g/L and 17.37 ± 0.09 g/L, respectively.

Previous research by Corsetti et al. [28] reported that *Fructilactobacillus sanfranciscensis* produces hexanoic acid, which has extremely high antifungal activity. Similarly, another study by Moroni et al. [29] investigated the strain *Fructilactobacillus sanfranciscensis* CB1, which produces organic compounds such as formic acid and n-pentanoic acid, and these compounds were found to have inhibitory effects on pathogenic bacteria in pasta products. Therefore, Ls5 has a higher TTA than Fs7, which can reduce the growth of spoilage microorganisms and positively impact the shelf-life of flour products.

#### 3.2.3. Morphostructural Characterization of LAB

The colonies of Ls5 and Fs7 on modified mMRS agar medium exhibited similar characteristics, as depicted in Figure 2D–G. The colonies were white or light yellow in color and exhibited a thicker, round, and clear-edged shape. Gram staining revealed a purple color, indicating that both organisms were Gram-positive, rod-shaped bacteria, existing singly, in pairs, or chains. Specifically, Ls5 had a size range of 1.30–1.60 μm × 2.5–8.0 μm, while Fs7 organisms measured 0.90–1.50 μm × 3.0–8.0 μm.

### 3.3. Comparison of EPS Characteristics Between the Two Strains

#### 3.3.1. Isolation and Purification of EPS from LAB

**Figure 3:**
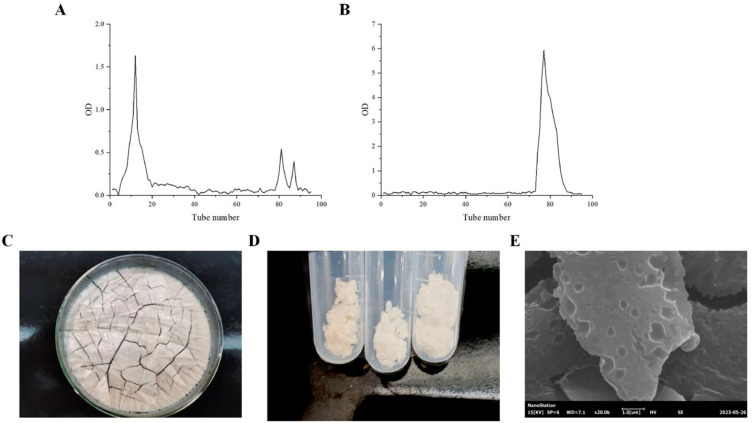
Elution curves of EPS Ls5 (**A**), Elution curves of EPS of Fs7 (**B**), Morphological characteristics of unpurified EPS (**C**), Morphological characteristics of purified EPS (**D**), Electron microscopy of EPS (**E**).

EPS produced by the two strains were isolated, purified, and characterized. The elution curves of EPS from the target strains are shown in Figure 3A,B. The collected sample solution was subjected to multiple concentration steps, including spinning, dialysis, and lyophilization, as shown in Figure 3C–E. The resulting EPS had a slightly yellowish color and a loose and porous structure. The further purified EPS was white, lightweight, and slightly sticky. Electron microscopy analysis of the purified EPS revealed a rough surface with numerous small, dense holes dispersed throughout.

#### 3.3.2. Ultraviolet Spectrum of EPS of LAB

**Figure 4:**
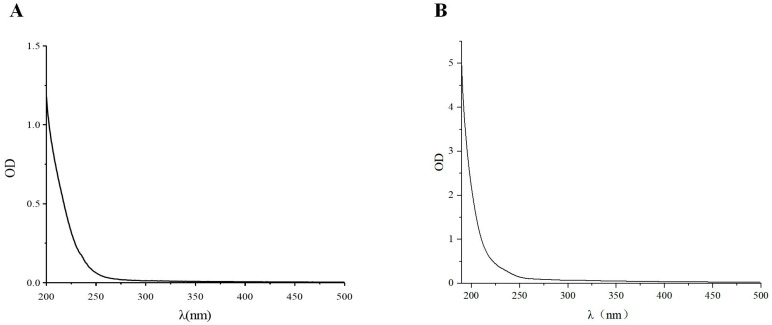
Ultraviolet spectra of Ls5 EPS (**A**), Ultraviolet spectra of Fs7 EPS (**B**).

As depicted in Figure 4A,B, purified EPS at both 260 and 280 nm were smooth and flat without any distinct peaks. This finding indicated that the sample contained minimal nucleic acids and proteins, suggesting a high level of purity.

#### 3.3.3. Monosaccharide Composition and Molecular Weight of EPS

**Figure 5:**
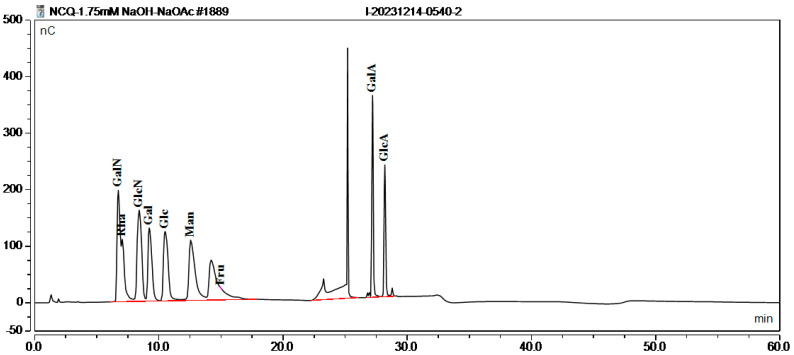
Standard curve of monosaccharides. Abbreviations in the figure represent the following monosaccharides: Galactosamine (GalN), Rhamnose (Rha), Glucosamine (GlcN), Galactose (Gal), Glucose (Glc), Mannose (Man), Galacturonic Acid (GalA), and Glucuronic Acid (GlcA).

**Figure 6:**
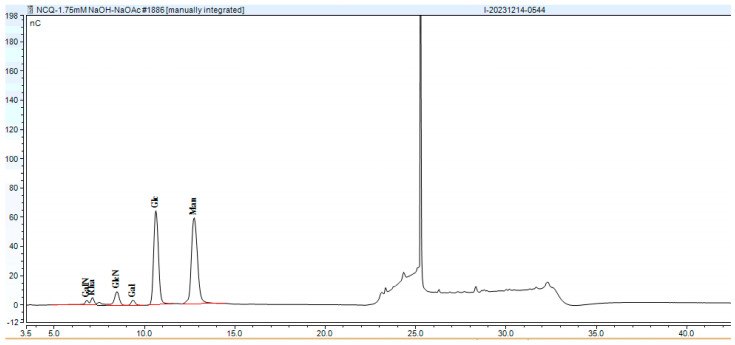
Monosaccharide composition of Ls5 EPS. Abbreviations in the figure represent the following monosaccharides: Galactosamine (GalN), Rhamnose (Rha), Glucosamine (GlcN), Galactose (Gal), Glucose (Glc), Mannose (Man), Galacturonic Acid (GalA), and Glucuronic Acid (GlcA).

**Figure 7:**
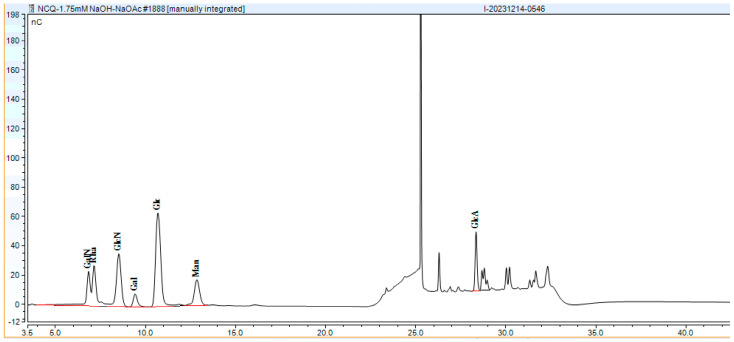
Monosaccharide composition of Fs7 EPS. Abbreviations in the figure represent the following monosaccharides: Galactosamine (GalN), Rhamnose (Rha), Glucosamine (GlcN), Galactose (Gal), Glucose (Glc), Mannose (Man), Galacturonic Acid (GalA), and Glucuronic Acid (GlcA).

The EPS produced by strains Ls5 and Fs7 were analyzed for monosaccharide composition and molecular weight, as depicted in Figure 5, Figure 6 and Figure 7. The EPS from both strains were identified as heteropolysaccharides. The EPS from Ls5 primarily comprised Glc and Man, with minor quantities of Glc and Man, and contained small amounts of GalN, Rha, GlcN, and Gal. The EPS from Fs7 predominantly contained Glc, GlcN, GalN, and Rha, with a small amount of Gal and Man. The molecular weight distributions are detailed in Table 2.

### 3.4. Comparison of pH, TTA and Fermentation Capacity of Different Sourdough

**Figure 8:**
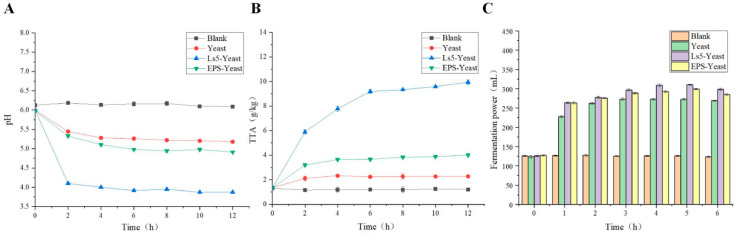
pH of different sourdough (**A**), TTA of different sourdough (**B**), Comparison of the fermentation capacity of different sourdough (**C**).

As illustrated in Figure 8A,B, over time, a discernible trend was observed in all four sourdough groups, where the pH initially decreased and then stabilized, whereas the total titratable acidity (TTA) showed a tendency to increase and then stabilize. The pH of sourdough samples ranged from 3.9 to 6.1, with TTA approximately at 1.2 ± 0.06, 2.3 ± 0.04, 9.9 ± 0.19, and 4.0 ± 0.13 g/kg, respectively. Notably, the Ls5-Yeast group exhibited the lowest pH and highest TTA among the sourdough samples. These findings suggest that LAB play a crucial role in acidifying dough, possibly by producing organic acids through microbial activity, lowering the pH to levels conducive to yeast growth and reproduction, utilizing yeast metabolites for their own growth and reproduction, and subsequently generating significant amounts of organic acids to enhance dough acidity [30]. Previous studies have indicated that the TTA of dough fermented with a combination of yeast and LAB is higher than that of dough fermented with yeast alone, with a notable difference in soluble sugar content [24]. Bastetti et al. [31] conducted a study on the impact of pH on sourdough quality and concluded that the optimal pH range for sourdough is between 3.7 and 4.1, which significantly enhances the flavor profile of flour products.

During dough rise, wheat proteins, specifically alcohol-soluble and gluten proteins, interact to form gluten networks. The viscoelasticity and air retention capacity of dough are largely determined by the development of the gluten network. Additionally, starch in dough forms cross-links with gluten proteins, which affect dough quality [32]. In Figure 8C, it can be observed that within the first three hours, the volume of sourdough containing Ls5 and EPS increased more significantly than that in the Yeast group. The volume of sourdough in the Ls5-Yeast group reached its peak at 310 mL after four hours but then decreased after five hours. Similarly, the volume of sourdough in the EPS-Yeast group reached its highest point of 300 mL after five hours and decreased after five hours. The rapid increase in sourdough volume in the Ls5-Yeast group was due to the ongoing acidification of the dough by Ls5 metabolism at the beginning of fermentation, which created an environment favorable for yeast proliferation. In contrast, the EPS-Yeast group showed more compact starch cross-linking because of the presence of EPS, which resulted in improved gas retention capacity and a significant increase in specific volume. This observation suggests that the addition of EPS has a beneficial effect on dough properties [32].

### 3.5. Comparison of pH and TTA of Different Mantou

**Figure 9:**
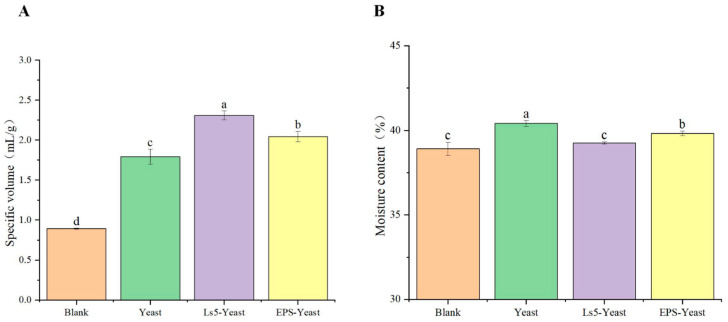
Specific volume of Mantou (**A**), Moisture content of Mantou (**B**). Lowercase letters indicate significant differences (*p* < 0.05) among samples. Different letters indicate significant differences.

The specific volume was significantly influenced by the gluten network structure and CO_2_ production level. As depicted in Figure 9A, the specific volume of Mantou produced from all groups, except the Blank group, met the national standard requirements (≥1.70 mL/g). Notably, the specific volume of Mantou in the Ls5-Yeast group surpassed that in the EPS-Yeast group, suggesting that the combined fermentation of LAB and yeast could positively affect the specific volume of Mantou. Previous studies have suggested that the concurrent fermentation of LAB and yeast may enhance CO_2_ production [33], thereby improving the fermentation properties of dough and increasing the specific volume of the Mantou. In addition, the incorporation of EPS enhanced dough leavening performance and facilitated the formation of a macromolecular polymer from starch and protein.

The moisture content of the Mantou varied among the four groups, with the Yeast group exhibiting the highest moisture content at 40.4 ± 0.2%, followed by the EPS-Yeast, Ls5-Yeast, and Blank groups, as shown in Figure 9B. The difference in moisture content among the groups can be attributed to the lower microbial content in the Blank group, resulting in limited water production by microorganisms. During the fermentation process, yeast participates in aerobic respiration, leading to the generation of water, which exceeds water consumption [30], thereby resulting in a higher water content in the Mantou of the Yeast group. Conversely, the water content in the Mantou of the Ls5-Yeast group decreased because of the production of EPS and other byproducts by LAB through water metabolism. Despite the acidification of the dough and the promotion of yeast growth and metabolism by LAB, water content remained low. The impact of EPS on the moisture content of the Mantou may depend on the quantity of EPS added. A small amount of EPS may not significantly alter the moisture content of Mantou but may enhance moisture migration during storage.

### 3.6. Comparison of Shelf-Life of Different MANTOU

#### 3.6.1. Changes in Specific Volume During Storage

The specific volume variations of different Mantou during storage are presented in Table 3 and Table 4. The results indicate that the specific volumes of the four types of Mantou remained relatively constant throughout the storage period, regardless of whether they were stored at room temperature or 4 °C. This stability in a specific volume can be attributed to the partial dispersion of moisture despite the Mantou being stored in a sealed container. The dispersion of moisture facilitated the surface drying of the Mantou, contributing to a decrease in volume. In addition, moisture dispersion reduced the mass of the Mantou. Notably, alterations in the specific volume were not significantly pronounced [34].

#### 3.6.2. Changes in Moisture Content During Storage

**Figure 10:**
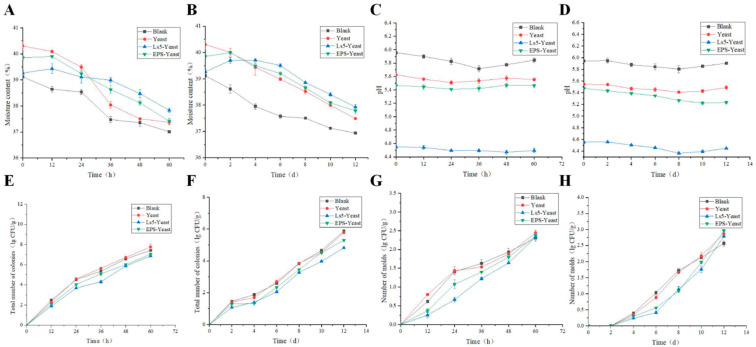
Moisture changes of Mantou at room temperature (**A**), Moisture change of Mantou at 4 °C (**B**), pH change of Mantous at room temperature (**C**), pH change of Mantou at 4 °C (**D**), The total number of colonies in Mantous changed at room temperature (**E**), The total number of colonies in Mantous changed at 4 °C (**F**), Changes in the number of molds in Mantous at room temperature (**G**), Changes in the number of molds in Mantous at 4 °C (**H**).

The moisture content of Mantou is an essential quality parameter. The moisture levels of the four groups of Mantou decreased over time at room temperature and 4 °C, as illustrated in Figure 10A,B. At the end of storage, the water content of Mantou in the Blank, Yeast, Ls5-Yeast, and EPS-Yeast groups decreased by 5.39, 7.27, 3.64, and 6.1%, respectively, at room temperature. Similarly, at 4 °C, the water content of the Mantou in the Blank, Yeast, Ls5-Yeast, and EPS-Yeast groups decreased by 5.57%, 7%, 3.41%, and 5.17%, respectively. The EPS-Yeast group exhibited a slower water loss rate, potentially due to hydrogen bonding between the EPS and water molecules, which enhanced the water-holding capacity and reduced water evaporation. On the other hand, the Ls5-Yeast group showed slower water loss due to LAB fermentation, which can degrade starch, alter its structure, increase amylose content, and enhance starch polymerization. This process leads to roughening of the starch particle surface, thereby increasing the surface area for water absorption and retention [21]. A key factor affecting water retention is the binding of water to starch within the starch gel network structure [35]. As storage progresses, the spatial arrangement of starch can become denser, leading to a corresponding reduction in the starch moisture content and decreased water retention [36]. Fik et al. [37] investigated the relationship between starch and water retention during storage, and their findings were consistent with the results of this study.

#### 3.6.3. pH Changes During Storage

As depicted in Figure 10C,D, the pH levels of the four categories of Mantou exhibited comparable trends, characterized by an initial decrease followed by an increase during storage at room temperature or under refrigeration. The growth and metabolic activities of microorganisms play a significant role in determining the pH of the noodle products. The combination of moisture depletion and bacterial growth can result in the production of acidic compounds in the Mantou, leading to a decrease in pH during the pre-storage phase [38]. Consequently, the microorganisms break down the proteins present in the Mantou, generating amines, ammonia, and other substances, which ultimately contribute to an increase in pH.

#### 3.6.4. Microbial Changes During Storage

Graphs depicting the total colony counts of Mantou stored at room temperature and refrigerated conditions over time are presented in Figure 10E,F. Initially, the total colony count of the Mantou cooled for 1 h after steaming was zero. Thereafter, the total colony counts of all four Mantou groups showed an upward trend. Specifically, when Mantou was stored at room temperature, the total colony count increased rapidly from 0 to 24 h. Between 24 and 60 h, the total colony count of Mantou in the Yeast group was the highest, whereas the total colony count of Mantou in the Ls5-Yeast group remained consistently lower than that of the other three groups. However, when Mantou was stored under refrigerated conditions, the total colony counts in the Ls5-Yeast group were lower than those in the EPS-Yeast, Yeast, and Blank groups. A previous study on flour Mantou [14] suggested that the total colony count of Mantou should not exceed 10^6^ CFU/g, which is equivalent to 6 log CFU/g. Notably, the total colony count of Mantou in both the EPS-Yeast and Ls5-Yeast groups did not surpass 10^6^ CFU/g within 12 days.

Figure 10G, H depict the changes in mold counts observed in the four groups at room temperature and during refrigerated storage. At room temperature, mold growth was remarkably rapid, exceeding the standard mold count within 60 h in all four groups. In contrast, during refrigerated storage, no molds were detected in the Mantou within the initial 0–2 days, suggesting that the lower temperature inhibited mold growth. Between 2–6 days, mold growth rates were slower, with the Blank group exhibiting the highest mold counts and the Ls5-Yeast group showing the lowest. As the storage time increased, mold growth rates accelerated in the Ls5-Yeast and EPS-Yeast groups. By day 12, all four types of Mantou displayed visible mold signs on their surfaces. According to flour Mantou [14], the acceptable limit for molds is ≤200 CFU/g, equivalent to 2.3 lg CFU/g. By day 12, the mold counts in all four groups had exceeded this threshold. Mold growth on the Mantou surfaces was more pronounced in the EPS-Yeast group. Therefore, Mantou from the Yeast group should be consumed within 36 h at room temperature, whereas those from the EPS-Yeast and Ls5-Yeast groups should be consumed within 48 h. All four groups were consumed within 12 days at room temperature, and in cold storage, they were not stored for more than 10 days.

The proliferation rate of microorganisms is influenced by the environmental temperature. Studies have demonstrated that at temperatures of 20 °C or higher, there is an increase in the growth rate of the total colonies, with the count of Yeast group bread surpassing the standard within 48 h. Conversely, at 4 °C, microbial growth was slower, leading to a prolonged lag phase. These findings indicate that the ambient temperature has a significant effect on the shelf-life of a product. The total colony count and mold count of the Ls5-Yeast group bread were lower than those of the Yeast group bread stored at room temperature and refrigerated conditions. This difference can be attributed to the rapid acidification of raw materials owing to the production of organic acids during sourdough fermentation, which effectively inhibits the growth of other bacterial strains [39]. Additionally, the reduced colony and mold counts of EPS-Yeast group bread may be linked to the inherent antioxidant and antimicrobial properties of EPS, as well as its ability to retard starch aging [4,5].

### 3.7. Comparison of Starch Digestion In Vitro

#### 3.7.1. Comparison of In Vitro Digestible Reducing Sugar Content of Starch

**Figure 11:**
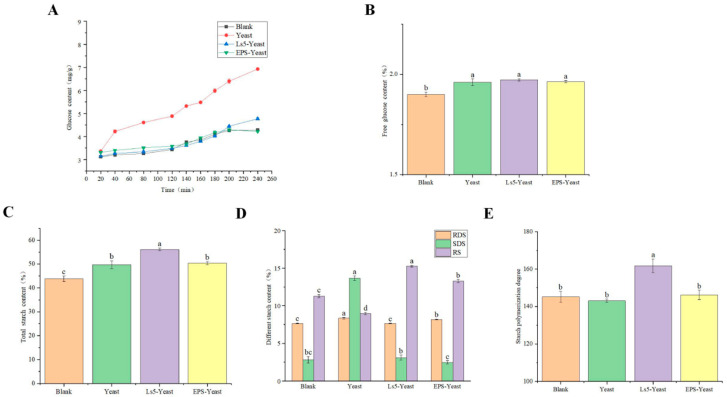
Yield of reducing sugar digested in vitro of Mantous (**A**), Free glucose content of Mantous (**B**), Total starch content of Mantous (**C**), RDS, SDS, and RS contents of different Mantous (**D**), Degree of starch polymerization of different Mantous (**E**). Lowercase letters indicate significant differences (*p* < 0.05) among samples. Different letters indicate significant differences.

Starch can be converted into glucose through the action of amylase during digestion, thereby indicating the in vitro digestion properties of starch-based on the changes in glucose content. The graphs in Figure 11A illustrate the variations in glucose content in the Mantou of the Blank, Yeast, Ls5-Yeast, and EPS-Yeast groups over a 240-min period.

During the initial 20 min of digestion, no discernible difference in the reducing sugar content was observed among the four groups. However, during the gastric digestion phase (20–120 min), a notable difference in reducing sugar content resulting from starch digestion was observed, with the Yeast group of Mantou exhibiting the highest glucose content and the Blank group of bread showing the lowest. In the subsequent intestinal digestion phase (120–240 min), the reducing sugar content was ranked in the following order: Yeast group of Mantou had the highest content, followed by EPS-Yeast group, Blank group, and Ls5-Yeast group. Notably, the reducing sugar content of Ls5-Yeast group bread was consistently lower than that of EPS-Yeast group bread in the early stages; however, at 200 min, the reducing sugar content briefly increased and surpassed that of the EPS-Yeast group and Blank group bread. At the end of digestion, the Yeast group bread had the highest glucose content, while the EPS-Yeast group had the lowest. These findings suggest that the incorporation of *Fructilactobacillus sanfranciscensis* Ls5 and its EPS can retard starch digestion and mitigate postprandial blood glucose elevation. This effect may be attributed to the presence of Ls5 during fermentation, which reduces the environmental pH and inhibits amylase activity. Additionally, EPS can interact with starch to form complexes and encapsulate starch granules, thereby preventing amylase from breaking down starch granules.

#### 3.7.2. Comparison of Free Glucose and Total Starch Content

As depicted in Figure 11B, the mass fractions of free glucose (FG) in the Mantou from the Blank, Yeast, Ls5-Yeast, and EPS-Yeast groups were 1.9 ± 0.01%, 1.96 ± 0.01%, 1.97 ± 0.01%, and 1.96 ± 0.01%, respectively. Moreover, Figure 11C reveals that the total starch mass in the Mantou of the Blank, Yeast, Ls5-Yeast, and EPS-Yeast groups were 43.8 ± 1.14%, 49.7 ± 1.6%, 56.2 ± 0.6%, and 50.39 ± 0.6%, respectively. Notably, the Ls5-Yeast group exhibited the highest total starch content, and the EPS-Yeast group displayed a slightly higher value than that of the Yeast group.

#### 3.7.3. Comparison of RDS, SDS, and RS Content and Starch Polymerization

This study aimed to investigate the impact of various types of starch on blood glucose levels. The results indicated that rapidly digestible starch (RDS) led to a significant increase in blood glucose, whereas resistant starch (RS) had a positive effect by moderating the postprandial rise in blood glucose and maintaining stability [22]. Figure 11D shows that the RDS content among the four groups was relatively consistent, with the soluble dietary fiber (SDS) content in the Mantou supplemented with Ls5 and EPS being 23% and 18% of the Yeast group, respectively. In contrast, RS content increased by 70% and 48% in the same groups. The uniform RDS content across samples may be attributed to the fluffy Mantou structure in the Ls5-Yeast and EPS-Yeast groups, which enhances amylase accessibility and facilitates starch hydrolysis [40]. The variations in SDS and RS contents suggested that the presence of Ls5 and EPS in the Mantou had an inhibitory effect on starch digestion, leading to the formation of resistant starch. This phenomenon was associated with the amylopectin structure (branched and straight-chain), protein fractions, and starch dextrinization of the samples.

One of the key factors contributing to the formation of resistant starch is the degree of polymerization of starch molecules and the amylose content in food [41]. The degree of polymerization of the four Mantou categories was evaluated, as shown in Figure 11E. The average degree of polymerization of Mantou in the Ls5-Yeast group was higher than that in the Yeast group (161.7), whereas the starch polymerization degree of Mantou in the EPS-Yeast group was slightly higher than that in the Yeast group (146.2), which is consistent with the findings of Eerlingen et al. [42,43]. Several factors can contribute to the increase in the degree of polymerization. During fermentation, LAB reduce the levels of protein, fat, and other nutrients in the dough, increase amylose content, and enhance the degree of starch polymerization. Second, LAB fermentation facilitates the breakdown of the long amylopectin chains in the dough, leading to the rearrangement of decomposed branch chains into linear chains, consequently elevating the amylose content and the degree of starch polymerization [35].

### 3.8. Sensory Evaluation

**Figure 12:**
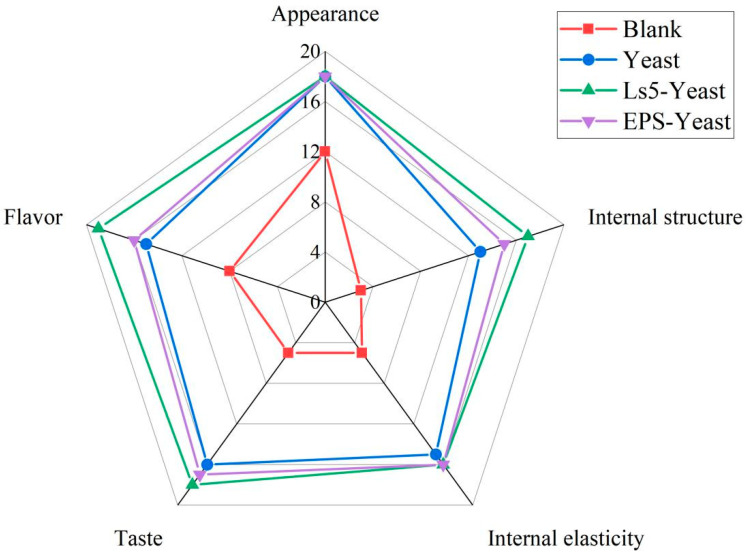
Sensory evaluation of Mantou.

Ten food experts evaluated the sensory attributes of Mantou from four different groups: Blank, Yeast, Ls5-Yeast, and EPS-Yeast groups. Figure 12 indicated that the Mantou from the Ls5-Yeast group received the highest score of 88 points, followed by the EPS-Yeast group with a total score of 82 points, Yeast group with 77 points, and Blank group with the lowest score of 32 points.

The visual characteristics of Mantou in the Yeast, Ls5-Yeast, and EPS-Yeast groups showed no significant differences. However, the internal structure of the Mantou in the three groups exhibited notable differences. The texture of the Yeast group was coarser than those of the other two groups, with slightly rougher stomata. Despite this, all three Mantou groups were deemed to have good taste. Ls5-Yeast and EPS-Yeast groups were particularly praised for their delicate texture, elasticity, and pleasant mouthfeel. Notably, the most prominent disparity among the groups was observed in the flavor profile of Mantou. The Ls5-Yeast group stands out for its superior flavor, which is characterized by a sweet taste and robust wheat aroma. In contrast, the variance in flavor between the Mantou of the EPS-Yeast group and the Yeast group was minimal. This suggests that while EPS significantly improved the texture of the Mantou, its impact on flavor enhancement was relatively modest.

## 4. Conclusions

In this study, LAB with high EPS production were screened from LAB obtained from sourdough samples, and the target strains Ls5 and Fs7 were identified. The exopolysaccharides produced by LAB and their role in the fermentation process of sourdough, as well as their potential impacts on the quality and characteristics of Mantou, were studied. The incorporation of Ls5 and its EPS into Mantou resulted in a lower dough fermentation pH, increased organic acid production, and enhanced fermentation activity. Additionally, the incorporation of Ls5 and its EPS led to significant improvements in the quality of the Mantou, including the extension of shelf-life, improved sensory evaluation, and a reduction in sugar content. There was an increase in resistant starch content during digestion in both types of Mantou, which could offer potential benefits to human glycemic health. This study can provide some technical guidance for the improvement of the traditional Mantou production process, enhancing its physical and chemical properties and taste, and help to promote the modernization and innovation of traditional food technology.

## Figures and Tables

**Table 1:** The amount of growth of the target strain.

Parallel Experimental Group	1	2	3	Mean ± SD
Ls5 growth amount/g·L^−1^	2.6	2.64	2.64	2.62 ± 0.02
Fs7 growth amount/g·L^−1^	2.24	2.28	2.24	2.26 ± 0.02

**Table 2:** Molecular weight of EPS. (Number-average molecular weight—Mn, peak-position molecular weight—Mp, viscosity average molecular weight—Mv, weight-average molecular weight—Mw, Z-average molecular weight—Mz, and polydispersity—Mw/Mn or Mz/Mn).

	Mn (Da)	Mp (Da)	Mv (Da)	Mw (Da)	Mz (Da)	Mw/Mn	Mz/Mn
Ls5 EPS	3.34 × 10^4^	4.57 × 10^4^	5.26 × 10^4^	5.96 × 10^4^	1.35 × 10^5^	1.787	4.043
Fs7 EPS	2.23 × 10^4^	1.94 × 10^4^	3.06 × 10^4^	3.45 × 10^4^	9.01 × 10^4^	1.549	4.046

**Table 3:** Specific volume changes of Mantou at room temperature.

Time	Blank Group	Yeast Group	Ls5-Yeast Group	EPS-Yeast Group
0 h	0.89 ± 0.01 ^a^	1.8 ± 0.01 ^ab^	2.34 ± 0.03 ^a^	2.01 ± 0.04 ^a^
12 h	0.86 ± 0.01 ^a^	1.82 ± 0.01 ^a^	2.22 ± 0.13 ^ab^	1.98 ± 0.01 ^a^
24 h	0.86 ± 0.041 ^a^	1.82 ± 0.01 ^a^	2.09 ± 0.09 ^b^	1.95 ± 0.02 ^a^
36 h	0.84 ± 0.05 ^a^	1.79 ± 0.01 ^ab^	2.2 ± 0.09 ^ab^	1.96 ± 0.01 ^a^
48 h	0.85 ± 0.01 ^a^	1.77 ± 0.02 ^b^	2.06 ± 0.09 ^b^	1.98 ± 0.02 ^a^
60 h	0.82 ± 0.02 ^a^	1.78 ± 0.03 ^ab^	2.22 ± 0.06 ^ab^	1.96 ± 0.01 ^a^

Note: Different superscript letters indicate significant differences (*p* < 0.05).

**Table 4:** Specific volume change of Mantou at 4 °C.

Time	Blank Group	Yeast Group	Ls5-Yeast Group	EPS-Yeast Group
0 d	0.89 ± 0.01 ^a^	1.8 ± 0.01 ^bc^	2.34 ± 0.03 ^a^	2.01 ± 0.04 ^a^
2 d	0.86 ± 0.01 ^bc^	1.84 ± 0.02 ^ac^	2.2 ± 0.02 ^abc^	1.97 ± 0.02 ^ab^
4 d	0.87 ± 0.01 ^b^	1.81 ± 0.01 ^bc^	2.15 ± 0.05 ^bc^	1.95 ± 0.02 ^ab^
6 d	0.86 ± 0.004 ^bc^	1.88 ± 0.03 ^a^	2.27 ± 0.01 ^ab^	1.96 ± 0.01 ^ab^
8 d	0.87 ± 0.01 ^ab^	1.85 ± 0.02 ^ab^	2.15 ± 0.02 ^bc^	1.96 ± 0.01 ^ab^
10 d	0.85 ± 0.01 ^c^	1.8 ± 0.02 ^c^	2.11 ± 0.12 ^c^	1.94 ± 0.03 ^b^
12 d	0.85 ± 0.01 ^bc^	1.82 ± 0.01 ^bc^	2.13 ± 0.08 ^bc^	1.95 ± 0.02 ^ab^

Note: Different letter superscripts indicate significant differences (*p* < 0.05).

## Data Availability

The original contributions presented in the study are included in the article, further inquiries can be directed to the corresponding authors.
